# Supplementary material for: Cell-like pressure sensors reveal increase of mechanical stress towards the core of multicellular spheroids under compression
Source: Nat Commun. 2017 Jan 27;8:14056. doi: 10.1038/ncomms14056 (PMC5290143; doi:10.1038/ncomms14056)
Supplement: Supplementary Information — Supplementary Figures, Supplementary Notes and Supplementary References. [file ncomms14056-s1.pdf]

Supplementary Note 1 - Diffusion of fluorescent tracers within PAA microbeads

In order to define the bulk modulus of PAA microbeads, we used 1.5-2 MDa Dextran to apply an isotropic stress. The stress can be adjusted by the concentration of the Dextran polymer who has to necessarily be bigger than the exclusion pore size of the PAA gel. We therefore used fluorescent molecules to observe the penetration and estimate the exlusion pore size. Supplementary Figure 1 shows that sulpho rhodamine B infuses and concentrates within beads. Bigger dextran tracers 40 and 70 kDa do not enter the volume of the bead suggesting that the exclusion pore size is smaller than ~8.9 nm. Therefore, application of 2 MDa dextran (ϕ ≈ 54 nm) that is significantly bigger than 40 kDa and 70 kDa, can serve to compress beads by an osmotic stress in a controllable manner without a risk of partial infusion into a gel. (Scale bar 20 µm)

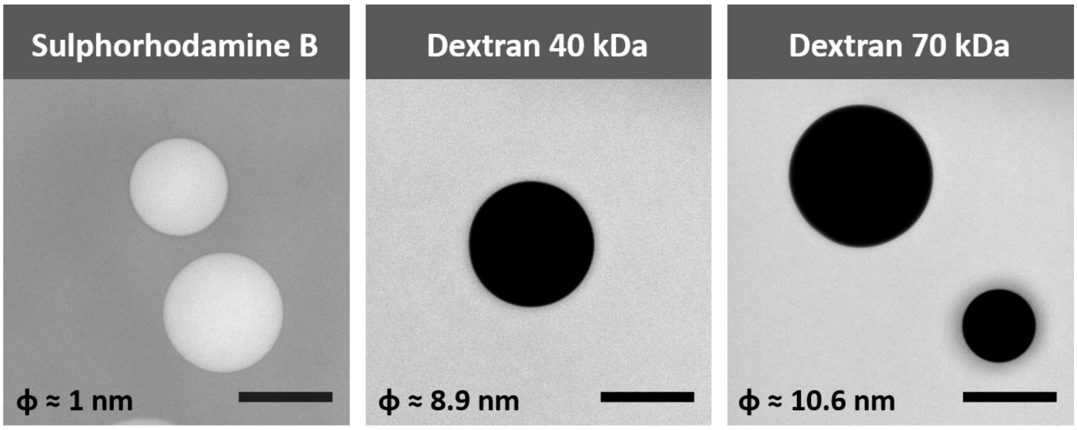

Supplementary Figure 1 Determination of the pore exclusion size of PAA beads. ϕ is an approximate hydrodynamic diameter. Scale bar: 40 µm

Supplementary Note 2 - Defocusing-associated indetermination of bead radius

The accuracy on bead radius measurements depends on precise determination of the equatorial plane of the bead. As the equatorial plane is determined by an empirical adjustment of the focal plane, it is worth quantifying the error associated to accidental defocusing.

Supplementary Figure 2 [left] shows the variations of the measured radius, when the bead is progressively defocused below (negative values) or above (positive values) the equatorial plane. The bead radius is expressed in percentage of the radius measured at the equatorial plane. The plot indicates that the error does not exceed ±0.5%, within a range of 30 µm. Supplementary Figure 2 [right] shows how the bead appears when the microscope focuses on the equatorial planes (above) and when defocused by more than 15µm (below). In practice, the defocusing is visible as it exceeds 6µm. In this range, experimental fluctuations dominate over the indetermination due to a bad estimation of the equatorial plane.

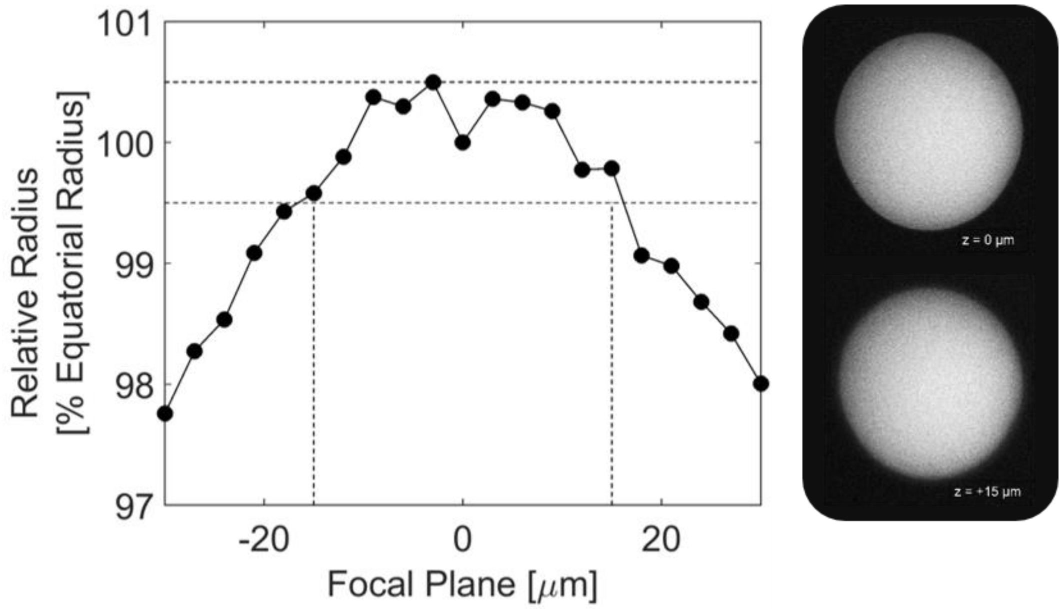

Supplementary Figure 2 [Left] Relative radius according to the focal plane. [Right] FITC images of the same bead at different focal planes.

Supplementary Note 3 - Image analysis – method of image thresholding

Image analysis has been based on the observation of the surface area of the particular bead before and after external application of compressive stress to CT26 spheroids. Size of the spheroid has been calculated from the manually chosen polygon selections. Ratio of background to intensity of the beads has varied in between different spheroids and therefore, we decided to determine area of beads using an automated algorithm of threshold [1]. Briefly, the procedure first divides the image into object and background by taking an initial threshold. The average of the pixels from both, the background and the object, are calculated. The algorithm continues to calculate both averages for sequentially incremented threshold until the threshold is larger than the composite average. Isodata threshold has been applied to images using imageJ software. Background for threshold has been limited by a predefined ROI. However, in cases were beads were found close to each other, another ROI has been adjusted in order to eliminate signal coming from the neighbouring beads. We have plotted the strain (ΔV/V) vs the fixed threshold increasing from 10 to 90 % to show that the variation of the strain is small in the range of 45-55% of the threshold (Supplementary Figure 3A). We have estimated that the maximal error of measured strain is ±0.018 as has been calculated by the difference of strain for fixed threshold at 45% and 55% (region depicted by dotted lines on Supplementary Figure 3A). We performed also a control analysis with the threshold fixed at 50% for all beads before and after, and we have found that in both cases we observe an increasing strain along the profile, with points being at a distance not bigger than the maximal error (Supplementary Figure 3B).

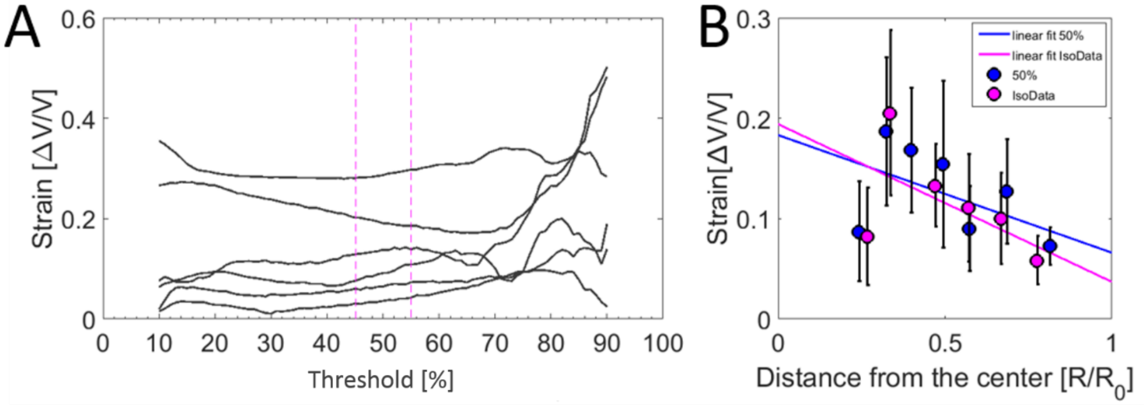

Supplementary Figure 3 A) Strain variation upon different pre-fixed tresholds. Magenta dotted lines determine the region within which IsoData algorithm has defined the threshold. B) Comparison of analysis performed at 50% of threshold (blue) and threshold that varied in the function of the background (isodata, Magenta).

Supplementary Figure 4 - Centrosome orientation within spheroids

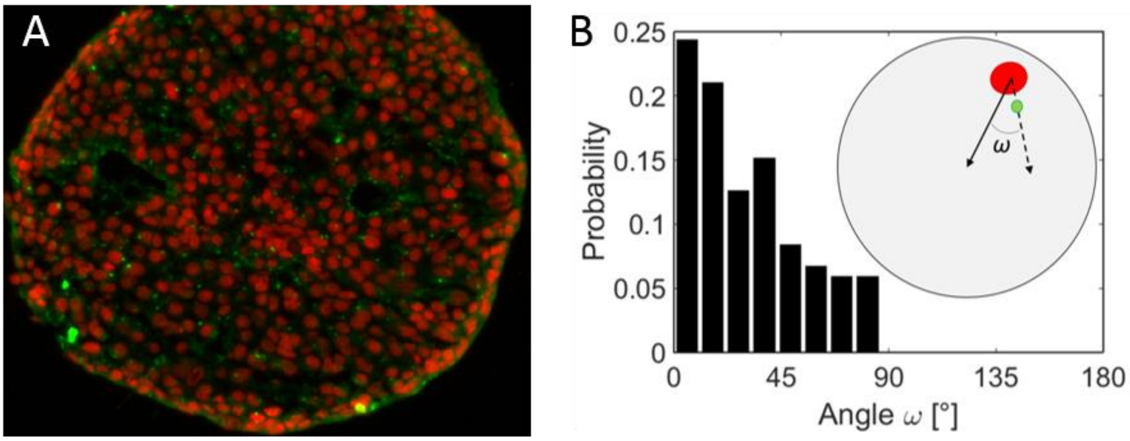

Supplementary Figure 4 Cell polarization in spheroids made of BC52 cells. A) Cryosection of a spheroid with nuclei stained by Hoechst (red) and centrosome sainted in green. B) Orientation probability as a functi on of the angle between nucleus/centrosome axis and nuclei/spheroid center axis. The graphs shows that the nucleus-centrosome axis is preferentially oriented toward the spheroid core

Supplementary Note 4 - ROCK inhibition changes the response to compressive stress

Rho-associated protein kinase (ROCK) in non-muscle cells regulates actin-cytoskeleton assembly and cell contractility. We used a Y-27632 inhibitor (at 10 μM) of ROCK in order to study the importance of the cytoskeletal activity on the propagation of the pressure within spheroids exposed to a compressive stress.

ROCK inhibition stopped spheroids growth and the volume of observed spheroids remained at a constant level (during 1 hour of observation after the drug has been added) in contrast to the control (Supplementary Figure 5a). Interestingly, inhibition of ROCK did not change the global spheroids compression, which was 8.9% for a control, and 8.8% for Y-27632 treated spheroids (non-significant difference, N=50 control and N=53 for Y27632, p=0.9639) (Supplementary Figure 5b). Supplementary Figure 5c presents the change of measured beads strain along the radius upon spheroids compression. We observed that inhibition of ROCK modifies the stress propagation within the spheroids. In the treated spheroids, the strain has been noticeably higher by the border (0.6-1 of the radius) and achieved levels comparable to the control by the center of spheroids. Our results point out that ROCK inhibition had visible influence on cells situated by the border, which as shown previously, are the ones that are biologically active and continue to proliferate in the growing spheroids. Our results further suggest that proliferation itself can be responsible for the accumulation of cellular anisotropy within the spheroids (as previously proposed[2]), which in turn has an influence on the propagation of the pressure.

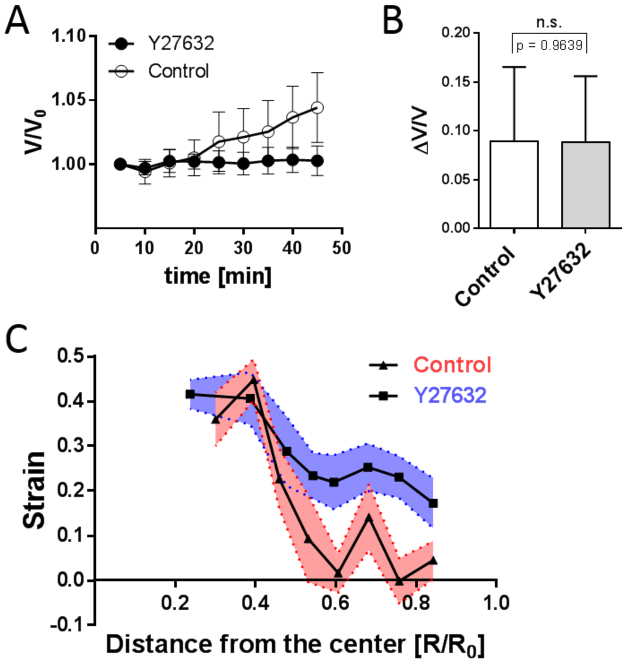

Supplementary Figure 5 ***A)*** *Evolution of spheroids size during 1 hours after treatment with Y27632 and in a control (N=8 spheroids for Y27632, and N=7 for the control). Error bar +/- SD* ***B)*** *Comparison of a global compression of spheroids non-treated (N=50) and treated with 10µM Y27632 (N=53). Error bar +/- SEM. Statistical test – t-test with p=0.0639.* ***C)*** *Measured strain in the function of the position within the spheroid (0 –core and 1 - border of the spheroid) for spheroids treated with Y27632 (blue, N=135) and for the control (red, N=81). Data points are grouped together in bins, with the error bar being a S.E.M and the position being and average position within the bin.*

Supplementary References

1 Ridler, T. W. & Calvard, S. PICTURE THRESHOLDING USING AN ITERATIVE SELECTION METHOD. *Ieee Transactions on Systems Man and Cybernetics* **8**, 630-632 (1978).

2 Delarue, M., Joanny, J.-F., Juelicher, F. & Prost, J. Stress distributions and cell flows in a growing cell aggregate. *Interface Focus* **4**, doi:10.1098/rsfs.2014.0033 (2014).
